# Answer to Gerber *et al.* “*Autosomal recessive pathogenic MSTO1 variants in hereditary optic atrophy*”

**DOI:** 10.15252/emmm.202216251

**Published:** 2023-07-11

**Authors:** Anikó Gál, Janine H Santos, Mária Judit Molnár, György Hajnóczky

**Affiliations:** ^1^ Institute of Genomic Medicine and Rare Disorders Semmelweis University Budapest Hungary; ^2^ Mechanistic Toxicology Branch, Division of the National Toxicology Program (DNTP) National Institute of Environmental Health Sciences (NIEHS), National Institutes of Health (NIH) Research Triangle Park NC USA; ^3^ MitoCare Center for Mitochondrial Imaging Research and Diagnostics, Department of Pathology, Anatomy and Cell Biology Thomas Jefferson University Philadelphia PA USA

## Abstract

Gal *et al* address the issues raised by Gerber *et al* and reiterate that patients in their study showed decreased Misato homolog 1 (MSTO1) mRNA and protein levels, but also confirm finding of Gerber *et al* that the mutation is in MSTO2p pseudogene. Whether MSTO2p variant contributes to the observed decrease in MSTO1 levels in patients remains unclear.

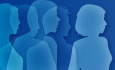

The correspondence article by Gerber *et al.* “*Autosomal recessive pathogenic MSTO1 variants in hereditary optic atrophy*” (Gerber *et al*, 2023) is questioning the disease relevance of the c.22G>A p. (p.(Val8Met) or V8M) variant in MSTO1 we reported in 2017. They identified the same rare variant in 16/49 patients having optic neuropathy in heterozygous form. Gerber *et al* argue that the identified variant is a relatively common variant of MSTO2p lncRNA n.83G>A (rs11264409) which shows very strong homology with the reported MSTO1 V8M (rs762798018) missense mutation. The minor allele frequency of the MSTO2p pseudogenic alteration is 0.326 compared to the reported mutation which is a rare variant of MSTO1 with a 0.000202 allele frequency.

We resequenced both Patient 1 (I/1) and 2 (II/1) samples with our original primer that does not discriminate between *MSTO1* and *MSTO2p*, and also with the *MSTO1* and *MSTO2p*‐specific primers introduced by Gerber *et al* (2023). Using the original primers, the patients again presented heterozygous *MSTO1* mutation but using the new primers they showed no mutation in *MSTO1* and homozygous mutation in *MSTO2p* (Fig 1).

**Figure 1:**
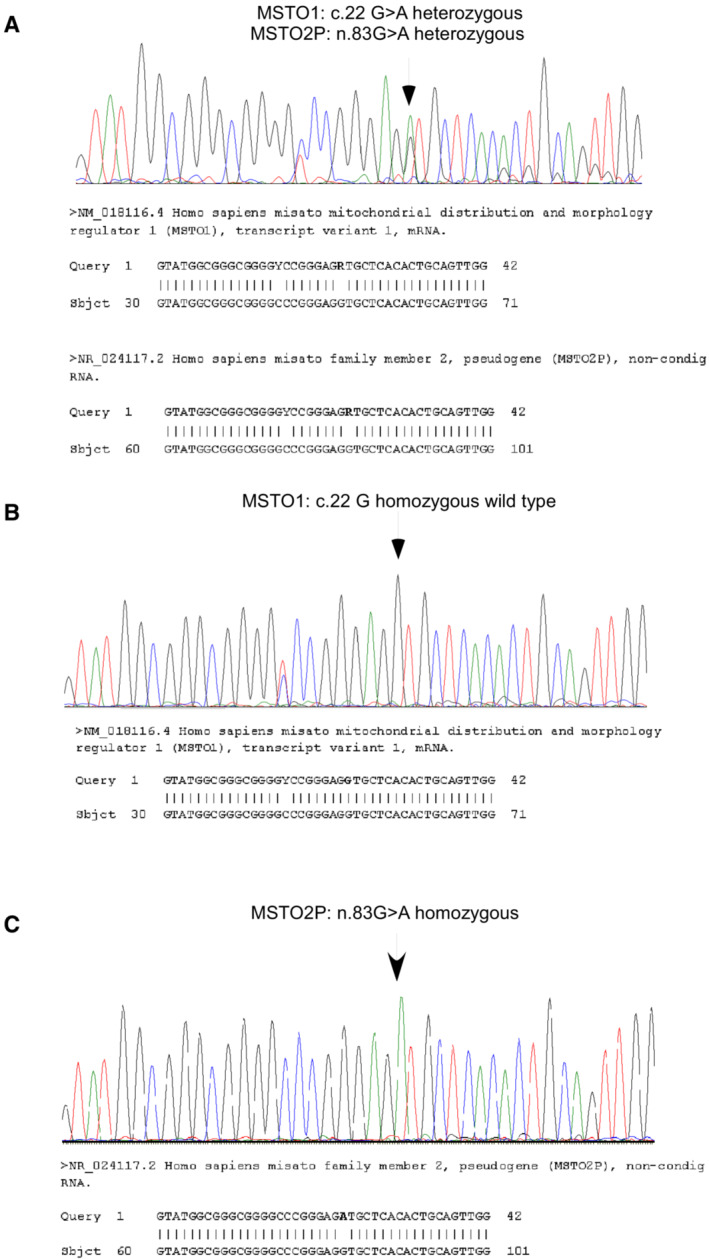
Sequencing results of exon 1 of *MSTO1* and *MSTO2P* using Sanger methodology (A) The results of the repeated Sanger sequencing with the original primers (Gal *et al*, 2017). As a result of the sequencing, a heterozygous c.22G>A (*MSTO1*) and a heterozygous n.83G>A (*MSTO2P*) variant was present in the investigated region. Due to a high homology the *MSTO1* and *MSTO2P* genes could not be separated. (B) *MSTO1* sequencing with the newly designed section‐specific primers. As a result of the new sequencing, in the 22^nd^ nucleotide position of the coding section of the. *MSTO1* gene the guanine is present on both alleles. (C) *MSTO2P* sequencing with the newly designed section‐specific primers. In the *MSTO2P* gene the n.83G>A variant is present in homozygous form.

Database information (https://www.ncbi.nlm.nih.gov/snp/rs11264409) and results by Gerber *et al* (2023) indicated that the present mutation in *MSTO2p* is common and likely benign. However, it is worth noting that both patients 1 (I/1) and 2 (II/1) in our original paper Gal *et al* (2017) showed decreased MSTO1 mRNA and protein levels (fig 1E and F) and a mitochondrial phenotype (figs 2 and 3). Importantly, we showed that genetic rescue of MSTO1 in the patient cells reversed the mitochondrial changes (fig 4) and that silencing of *MSTO1* in HeLa cells recapitulated the fusion defects observed in the patient cells (fig 5). Thus, changes in MSTO1 protein levels seem to contribute to the mitochondrial dynamics phenotype of the family.

The *MSTO2p* gene is transcribed but believed to be non‐coding. While it remains unclear which mRNA isoform (*MSTO1* or *MSTO2p*) is decreased in our patients, the diminished band in our western blots is reflective of loss of MSTO1 protein in the patient cells (Fig 1F; Gal *et al*, 2017).

The new analysis suggests that in our patients, mutations in *MSTO1* are not responsible for the decreased expression of the gene and protein, raising questions of how MSTO1 levels are regulated. One possibility would be that the homozygous mutation identified in *MSTO2p* make it act as a lncRNA that can regulate expression of MSTO1. In cancers, *MSTO2p* has been shown to work as a lncRNA that can regulate gene expression, including *EZH2* (Wei *et al*, 2017; Wang *et al*, 2019; Guo & Zhang, 2022). Notably, heterozygous c.553G>C p.(Asp185His) (rs2302427) variation of the *EZH2* gene was also detected in our Patients (I/1 and II/1). Based on this, the question arises as to whether the interaction of *MSTO2p* with another gene affects MSTO1 or mutations in a yet unidentified gene that can regulate MSTO1 levels is responsible for the phenotype in the family we reported previously (Gal *et al*, 2017).

Further studies will be necessary to validate if the homozygous *MSTO2p* mutation contributes or not to the decrease in MSTO1 mRNA and protein abundance identified in our patients. For example, quantification of *MSTO1* and *MSTO2p* mRNA levels using primers specific for each isoform can define if the decrease in *MSTO1* mRNA in patient fibroblasts reflects changes in one or both isoforms. Similarly, selective perturbations of MSTO1 and MSTO2p expression using siRNA or CRISPR can determine if *MSTO2p* might exert an effect on *MSTO1* RNA expression. Finally, characterization of the mitochondrial phenotype in patient cells upon decreases of MSTO2p or overexpression of wild‐type or the homozygous mutant will further shed light onto the role of this pseudogene in patients carrying *MSTO1* defects.

In summary, recent progress in discriminating of *MSTO2p* from *MSTO1* made it possible to reinterpret our original WES results as causative to the disease phenotypes of our patient population. However, the pathway we described – from decreased MSTO1 to mitochondrial dynamics and function in the patient fibroblasts – remains well supported and, as in our original conclusion, likely contributes to the patients' disease. Effectively, these new data now allows to extend our original results in trying to understand whether and how changes in MSTO2p might contribute to disease phenotypes associated with MSTO1 loss.
